# Solution-Processed PEDOT:PSS/MoS_2_ Nanocomposites as Efficient Hole-Transporting Layers for Organic Solar Cells

**DOI:** 10.3390/nano9091328

**Published:** 2019-09-16

**Authors:** Madeshwaran Sekkarapatti Ramasamy, Ka Yeon Ryu, Ju Won Lim, Asia Bibi, Hannah Kwon, Ji-Eun Lee, Dong Ha Kim, Kyungkon Kim

**Affiliations:** Deprtment of Chemistry and Nanoscience, Ewha Womans University, 52 Ewhayeodae-gil, Seodaemun-gu, Seoul 03760, Korea

**Keywords:** organic solar cells, MoS_2_, hole-transporting layer, oleylamine

## Abstract

An efficient hole-transporting layer (HTL) based on functionalized two-dimensional (2D) MoS_2_-poly(3,4-ethylenedioxythiophene):poly(styrenesulfonate) (PEDOT:PSS) composites has been developed for use in organic solar cells (OSCs). Few-layer, oleylamine-functionalized MoS_2_ (FMoS_2_) nanosheets were prepared via a simple and cost-effective solution-phase exfoliation method; then, they were blended into PEDOT:PSS, a conducting conjugated polymer, and the resulting hybrid film (PEDOT:PSS/FMoS_2_) was tested as an HTL for poly(3-hexylthiophene):[6,6]-phenyl-C_61_-butyric acid methyl ester (P3HT:PCBM) OSCs. The devices using this hybrid film HTL showed power conversion efficiencies up to 3.74%, which is 15.08% higher than that of the reference ones having PEDOT:PSS as HTL. Atomic force microscopy and contact angle measurements confirmed the compatibility of the PEDOT:PSS/FMoS_2_ surface for active layer deposition on it. The electrical impedance spectroscopy analysis revealed that their use minimized the charge-transfer resistance of the OSCs, consequently improving their performance compared with the reference cells. Thus, the proposed fabrication of such HTLs incorporating 2D nanomaterials could be further expanded as a universal protocol for various high-performance optoelectronic devices.

## 1. Introduction

Organic solar cells (OSCs) have many striking properties such as flexibility, solution processability, light weight, and simple manufacturing, especially if compared with their inorganic counterparts. To enhance their performance, numerous strategies have been proposed, including novel photoactive materials, morphology control, interfacial engineering, plasmonic nanoparticles incorporation, and alternative buffer layers and electrodes [1,2,3,4,5,6]. Their power conversion efficiency (PCE) has been recently improved up to >13% with rapid advances in new photovoltaic materials [7]. In the typical bulk heterojunction (BHJ) OSCs configuration, a photoactive blend layer consisting of acceptor/donor pairs is sandwiched between a bottom transparent anode and a top low-work-function cathode, combined with the corresponding interlayers. Such interlayers are crucial for determining the overall PCE and stability of OSCs because they reduce the potential energy barrier between photoactive layer and electrodes, enhancing the extraction of holes and electrons at the anode and cathode, respectively. 

Until now, many hole-transporting layer (HTL) materials, such as conducting conjugated polymers [8,9,10], conjugated polyelectrolytes [11,12] metal oxides/sulfides [13,14,15,16,17], and graphene oxide and its hybrid films [18,19,20,21], have been explored for use in OSCs. Among them, the conjugated polymer poly(3,4 ethylenedioxythiophene):poly(styrenesulfonate) (PEDOT:PSS) has been the most widely used due to its adequate work function for creating a good ohmic contact between active layer and anode, solution processability, and high conductivity. However, its hygroscopic and acidic nature often induces chemical instability between active layers and indium tin oxide (ITO) anodes, affecting the device stability and efficiency [22,23]. Moreover, there is a clear surface energy mismatch between PEDOT:PSS (hydrophilic nature) and the active layer (hydrophobic and made of, e.g., poly(3-hexylthiophene) (P3HT)) [24,25]. To overcome such drawbacks, various PEDOT:PSS modification strategies, such as incorporating metal nanoparticles [26,27,28], modification by metal salts [29,30], polymer doping [31,32], and hybridization with graphene [33,34], have been developed. Interfacial engineering with long alkyl chains is an alternative but attractive method to reduce the surface energy mismatch between HTL and active layer and also to accomplish desirable molecular orientation in the active layer for enhancing the charge transport in OSCs [35]. 

Single and few-layer molybdenum disulfide, a two-dimensional (2D) transition metal dichalcogenide (TMDC), has recently received much interest in electronics and optoelectronics research due to its excellent optical (bandgap: 1.8 eV), electrical (device mobility: 10–130 cm^2^ V^−1^ S^−1^), and mechanical (Young modulus: 270 GPa) properties [36,37]. Among the key preparation/exfoliation methods for TMDCs, namely, micromechanical cleavage [38], chemical vapor deposition [39], and liquid-phase exfoliation (LPE) [40], the latter is more attractive because it is scalable and cost-effective. MoS_2_ has been tested as HTL for OSCs [41,42,43] to exploit its extraordinary optical and electrical properties in photovoltaics; nevertheless, the results have revealed that neat MoS_2_ is not sufficient to replace PEDOT:PSS as OSC HTL, possibly because of its work function mismatch and unexpected phase transition. Hence, Xing et al. fabricated PEDOT:PSS/WS_2_ hybrid films and demonstrated their applicability as effective OSC HTLs [44]. However, the long-time (48 h) sonication they adopted for TMDC exfoliation in the PEDOT:PSS aqueous dispersion may affect the structure of both PEDOT:PSS and MoS_2_ in the final product; therefore, innovative strategies for effectively integrating these materials in OSCs are still highly demanded.

Here, we report the fabrication of oleylamine-functionalized MoS_2_ (FMoS_2_) combined with PEDOT:PSS as an effective hybrid HTL (PEDOT:PSS/FMoS_2_) for use in conventional P3HT:[6,6]-phenyl-C_61_-butyric acid methyl ester (PCBM)-based OSCs. The so-obtained OSCs exhibited better PCE and short-circuit current density (J_sc_) values compared with the reference cell having simple PEDOT:PSS as HTL. FMoS_2_ was characterized by various spectroscopic techniques including Raman spectroscopy, ultraviolet–visible (UV-Vis) absorption and transmittance, photoluminescence (PL), and transmission electron microscopy (TEM); the active layer microstructure and the surface properties of the hybrid HTL were analyzed by grazing-incidence wide-angle X-ray scattering (GIWAXS), atomic force microscopy (AFM), and contact angle measurements. Electrochemical impedance spectroscopy (EIS) measurements were carried out using an electrochemical analyzer (IVIUMSTAT.XR, IVIUM Technologies) under illumination at 0.1 V.

## 2. Experimental

### 2.1. Materials and Methods

The following chemicals were used in our experiment: molybdenum (IV) sulfide (˂2 µm, 99%) and oleylamine (Sigma-Aldrich, Gyeonggi-do, Korea), P3HT (1-Material, Gyeonggi-do, Korea), PEDOT:PSS (Heraeus Deutschland GmbH & Co., Leverkusen, Germany), isopropyl alcohol (IPA) (Dae-Jung Chemicals & Metals Co., Ltd., Gyeonggi-do, Korea), and methanol (Samchun Chemicals, Seoul, Korea).

### 2.2. Synthesis of FMoS_2_ Nanosheets and PEDOT:PSS/FMoS_2_ Hybrids

FMoS_2_ nanosheets were synthesized according to the liquid-phase exfoliation method reported in literature [45], with small modifications. Briefly, bulk MoS_2_ powder (200 mg) was bath-sonicated in oleylamine (2 mL) by using a Branson ultrasonic bath for 20 min and successively stirred at 60 °C for 12 h in an N_2_-filled glove box. Then, 1,2-dichlorobenzene (DCB) (18 mL) was added, and the dispersion was further bath-sonicated for 5 h. The resulting suspension was centrifuged at 4000 rpm, and the top 80% dark-green color supernatant, which contains excess oleylamine, DCB, and FMOS_2_ was collected. Then, the FMoS_2_ nanosheets were separated by adding excess acetone, followed by sonication for 2 min and high-speed centrifugation (10000 rpm). The separated FMoS_2_ nanosheets were settled at the bottom of the centrifuge tube, which was re-dispersed in a small amount of IPA by mild sonication, and different concentrations (5, 20, and 50 µL) of the resulting dispersion were added into PEDOT:PSS:methanol (1:1 V%) aqueous solutions, which were successively ultrasonicated for 30 min to obtain PEDOT:PSS/FMoS_2_ hybrid solutions.

### 2.3. Fabrication of OSCs

The OSCs having device architectures of ITO/PEDOT:PSS/P3HT:PCBM/LiF/Al and ITO/(PEDOT:PSS/FMoS_2_)/P3HT:PCBM/LiF/Al were fabricated as follows. ITO-coated glass substrates were cleaned via sequential ultrasonication in acetone, IPA, and distilled water, followed by oxygen plasma treatment for 10 min; then, they were spin-coated with a PEDOT:PSS (Clevios P VP Al 4083) or PEDOT:PSS/FMoS_2_ solution at 4000 rpm for 40 s and dried at 130 °C for 30 min to complete the HTL deposition. Next, an active layer consisting of a P3HT:PCBM (1:0.6 wt%) binary blend solution was spin-coated on the resulting HTL layer at 2500 rpm for 40 s inside an N_2_-filled glove box and annealed at 150 °C. Finally, LiF and Al layers were deposited by thermal evaporation. The active area of the fabricated OSCs was 0.06 cm^2^.

### 2.4. Characterization

The absorption properties of the samples were analyzed using a UV-Vis absorption spectrometer (Cary 5000, Varian, Inc.). Raman spectra were recorded on a Horiba Jobin-Yvon spectrometer. The emission properties were investigated with a luminescence spectrometer (LS55 Perkin Elmer). The TEM measurements were carried out on a JEOL JSM-2100-F system. The surface morphologies were investigated using a tapping-mode atomic force microscope (Veeco D3100). The water contact angles of the samples were measured with a KSV CAM 101 instrument. The GIWAXS analysis was conducted at the PLS-II 9A U-SAXs beamline of the Pohang Accelerator Laboratory (Korea) at the following operating conditions: incidence angle of ~0.12°, wavelength of 1.12 Å, and sample-to-detector distance of 224 nm. The GIWAXS patterns were recorded using a 2D charge-coupled device camera (Rayonix, SX-165, USA) with an exposure time of 10–30 s. The JV properties of the solar cells were measured with a Keithley 2400 solar cell IV measurement system under AM 1.5 G illumination at 100 mW cm^−2^.

## 3. Results and Discussion

**Figure 1:**
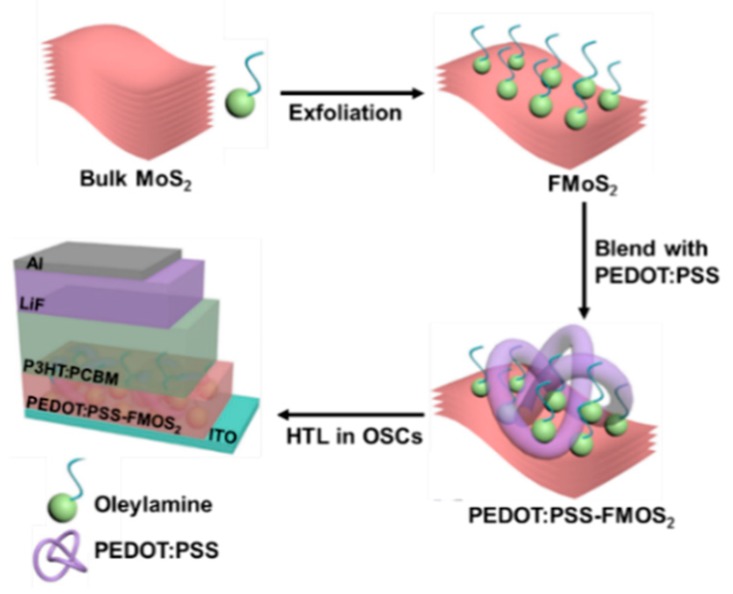
Fabrication process for poly(3,4-ethylenedioxythiophene):poly(styrenesulfonate) (PEDOT:PSS)/oleylamine-functionalized MoS_2_ (FMoS_2_) hybrid hole-transporting layer (HTL) for organic solar cells.

OSCs having two different device architectures, ITO/(PEDOT:PSS/FMoS_2_)/P3HT:PCBM/LiF/Al and ITO/PEDOT:PSS/P3HT:PCBM/LiF/Al (for comparison), were fabricated as schematized in Figure 1. 

First, we synthesized FMoS_2_ nanosheets via the solution-phase ultrasonic exfoliation of bulk MoS_2_ in the presence of oleylamine and 1,2-dichlorobenzene as a solvent; then, they were incorporated in different concentrations (5, 20, and 50 µL) into PEDOT:PSS, and the resulting PEDOT:PSS/FMoS_2_ (denoted as PEDOT:PSS/FMoS_2_(5), PEDOT:PSS/FMoS_2_(20), and PEDOT:PSS/FMoS_2_(50) according to the FMoS_2_ loading) was used as HTL for conventional OSCs.

**Figure 2:**
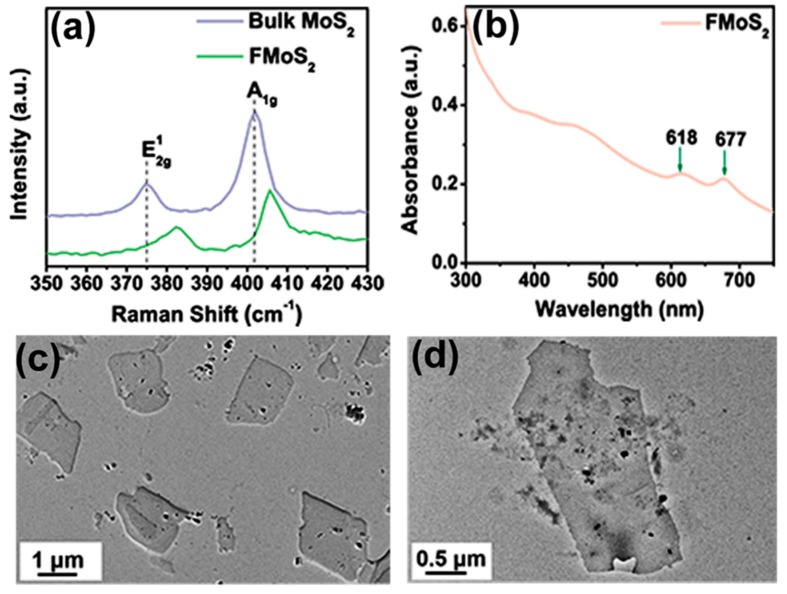
(**a**) Raman spectra of bulk and oleylamine-functionalized MoS_2_ (FMoS_2_). (**b**) Ultraviolet–visible light absorption spectrum of FMoS_2_. (**c**,**d**) Transmission electron microscopy images of FMoS_2_ nanosheets.

Raman spectroscopy is a powerful nondestructive technique for monitoring structural changes in 2D materials [46]. The Raman spectrum of bulk MoS_2_ showed two characteristic peaks at 374.83 and 402.05 cm^−1^ corresponding, respectively, to the E^¹^_2g_ and A_1g_ vibrational modes (Figure 2a); the first arose from the in-plane vibration of Mo and S atoms, while the second resulted from the out-of-plane vibrations of sulfur [47,48]. As regards FMoS_2_, the peaks for both the E^¹^_2g_ and A_1g_ vibrational modes were blue-shifted toward higher wavenumbers (respectively, 382.66 and 405.66 cm^−1^), suggesting interactions between oleylamine and MoS_2_. Moreover, the wavenumber difference between these two vibrational modes is closely related to the layer number present in the MoS_2_ nanosheets [49], and in our case, this difference decreased from 27.2 cm^−1^ for bulk MoS_2_ to 23 cm^−1^ for FMoS_2_ nanosheets, demonstrating the successful exfoliation of MoS_2_ nanosheets during the oleylamine treatment. 

The absorption properties of the FMoS_2_ nanosheets were further investigated via UV-Vis absorption spectroscopy; their spectrum (Figure 2b) clearly showed two characteristic absorption peaks of MoS_2_ at 618 and 677 cm^−1^ corresponding, respectively, to the A1 and B1 direct excitonic transitions with the energy split from valence band spin–orbital coupling [50]. Furthermore, unlike bulk MoS_2_, FMoS_2_ yielded dark-greenish dispersion in 1,2-dichlorobenzene. These results clearly indicate some alteration in the surface properties of MoS_2_ due to the oleylamine treatment [51].

Bulk MoS_2_ is an indirect bandgap semiconductor that does not exhibit any photoluminescence; however, upon exfoliation, its luminescence increases with decreasing its layer thickness, so that single-layer MoS_2_ shows the highest photoluminescence due to its transition into a direct bandgap semiconductor [52,53]. As expected, FMoS_2_ exhibited significant photoluminescence (see the PL spectra in Figure S1, Electronic Supporting Information (ESI)), which clearly proves the successful layer thinning of MoS_2_ during the functionalization process. 

The TEM images of the FMoS_2_ nanosheets are displayed in Figure 2c,d, showing a thin nanosheet morphology with sizes of several hundred nanometers. A careful observation of the nanosheet edges reveals the presence of few-layer nanosheets, confirming the effectiveness of the liquid-based exfoliation with oleylamine. AFM measurements were carried out (Figure S2, ESI) to further evaluate the layer thickness; that of FMoS_2_ was ~6.7 nm, suggesting the existence of few-layer nanosheets, while the reported thickness of monolayer MoS_2_ ranges between 0.9 and 1.2 nm [54].

To improve the performance of conventional PEDOT:PSS-based HTL for OSCs, we incorporated it with FMoS_2_ via a simple solution-blending method because we believed that the introduction of 2D sheet-like MoS_2_ functionalized with a long-chain primary alkyl amine (oleylamine) would have made the PEDOT:PSS surface more hydrophobic, facilitating the following deposition of the hydrophobic active layer. In addition, the amine group of oleylamine tends to be located near Mo atoms in MoS_2_ due to metal–amine interactions, while its long alkyl chain with –CH_3_ groups is oriented toward the active layer, and this kind of configuration should enforce the active layer with a desirable molecular orientation for efficient charge transport in OSCs; P3HT thin films deposited on insulator substrates modified with –CH_3_ groups formed face-on orientation because of π–H interactions [55,56].

The contact angles of ITO with PEDOT:PSS and PEDOT:PSS/FMoS_2_ containing 5, 20, and 50 µL of FMoS_2_ were 30°, 47°, 54°, and 56°, respectively (Figure S3, ESI), which indicates that the hydrophobicity of PEDOT:PSS was slightly increased by the FMoS_2_ addition and, hence, the hydrophobic active layer solution was more compatible on hybrid HTL than that of the hydrophilic PEDOT:PSS one.

The surface morphology of the various samples was compared via tapping-mode AFM analysis (Figure S4, ESI); the root-mean-square (rms) roughness value of PEDOT:PSS was 1 nm and decreased down to 0.69 nm for PEDOT:PSS/FMoS_2_(5), suggesting a smooth surface morphology in the hybrid HTL. However, PEDOT:PSS/FMoS_2_(50) exhibited an rms roughness value of 0.97 nm, indicating that the addition of higher FMoS_2_ concentrations would decrease the film smoothness. 

All the synthesized PEDOT:PSS and PEDOT:PSS/FMoS_2_ hybrid films exhibited similar UV-Vis transmittance values (Figure S5a, ESI), showing that the FMoS_2_ addition did not affect any absorption property of the PEDOT:PSS matrix. As regards the P3HT:PCBM (active layer) films spin-coated on glass substrates predeposited with PEDOT:PSS or PEDOT:PSS/FMoS_2_ HTLs (Figure S5b), for all the samples, their absorbance ranged from 400 to 650 nm, with a maximum at 512 nm, and two shoulders around 550 and 600 nm. The existence of vibronic feature at 600 nm suggests that the P3HT film existed in a high degree of ordered crystalline lamella due to strong interchain interactions [57].

**Figure 3:**
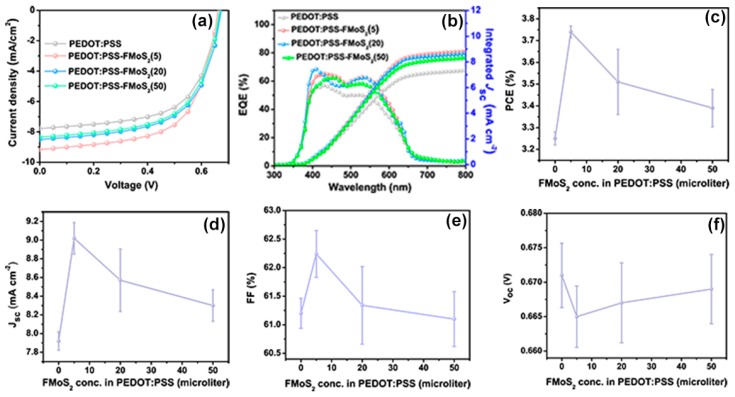
(**a**) Current density–voltage curves, (**b**) external quantum efficiency (EQE) profiles, (**c**) power conversion efficiencies (PCE), (**d**) short-circuit current density (J_sc_), (**e**) fill factor, and (**f**) open-circuit voltage (V_oc_) values of organic solar cells based on poly(3,4-ethylenedioxythiophene):poly(styrenesulfonate) (PEDOT:PSS) and PEDOT:PSS/oleylamine-functionalized MoS_2_ (FMoS_2_) as hole-transporting layers. The reported average PCE values are extracted from nine identical cells for each sample.

The current–voltage (JV) characteristics of the fabricated P3HT:PCBM OSCs having PEDOT:PSS/FMoS_2_ as HTL are shown in Figure 3a. Their performance is compared with that of reference devices having PEDOT:PSS as HTL in Table 1. The reference cells showed PCE = 3.25%, J_sc_ = 7.92 mA cm^−2^, V_oc_ = 0.671 V, and FF = 0.61. The FMoS_2_ incorporation led to significant PCE and J_sc_ improvements; in particular, the device based on PEDOT:PSS/FMoS_2_(5) exhibited the highest PCE, J_sc_, and FF. 

The external quantum efficiency (EQE) measurements (Figure 3b) showed improved EQE for the hybrid HTL-based OSCs compared with the reference cells and confirmed also their increased J_sc_, demonstrating the enhanced charge extraction at the HTL/active layer interface and the charge collection at the electrodes [58,59]. The photovoltaic parameters such as PCE, J_sc_, FF and V_oc_ as a function of FMoS_2_ in PEDOT:PSS HTLs are plotted in Figure 3c, d, e and f respectively.

**Figure 4:**
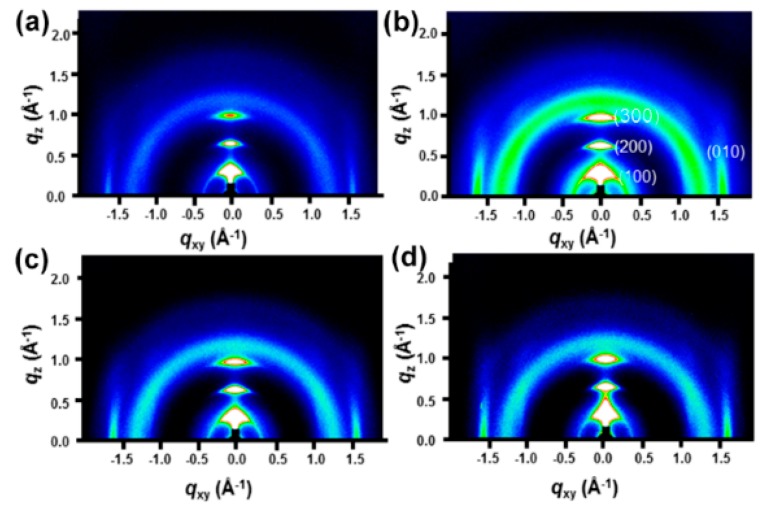
Grazing-incidence wide-angle X-ray scattering diffraction patterns of poly(3-hexylthiophene):[6,6]-phenyl-C_61_-butyric acid methyl ester thin films deposited on (**a**) poly(3,4-ethylenedioxythiophene):poly(styrenesulfonate) (PEDOT:PSS) and PEDOT:PSS combined with (**b**) 5, (**c**) 20, and (**d**) 50 µL of oleylamine-functionalized MoS_2_.

**Figure 5:**
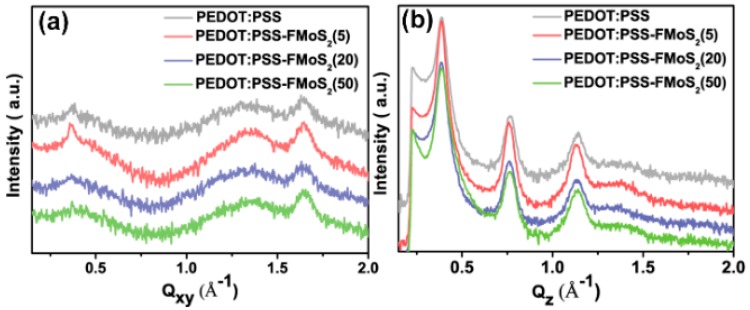
(**a**) In-plane and (**b**) out-of-plane spectra of poly(3-hexylthiophene):[6,6]-phenyl-C61-butyric acid methyl ester thin films deposited on poly (3,4-ethylendioxythiophene): poly(styrenesulfonate) (PEDOT:PSS) and PEDOT:PSS combined with 5, 20, and 50 µL of oleylamine-functionalized MoS_2_ samples obtained from grazing-incidence wide-angle X-ray scattering.

To understand the charge transport, we analyzed the microstructure (chain-orientation and crystallinity) of the active layer (P3HT:PCBM) on both the PEDOT:PSS and PEDOT:PSS/FMoS_2_ samples by GIWAXS (Figure 4 and Figure 5). Charge transport in conjugated polymers occurs either in the π–π staking direction or the chain backbone one, which is the fastest but its vertical alignment of chains backbones along the z direction is rarely observed [60,61]. In general, P3HT crystallizes into two main configurations, namely, edge-on and face-on orientations; in the former, both chain backbone and π–π staking directions lie parallel to the substrate; in the latter, π–π staking occurs perpendicular to the substrate, which is a desirable orientation in OSCs for vertical charge transport [62]. Figure 4 shows the GIWAXS diffraction patterns of P3HT:PCBM thin films deposited on PEDOT:PSS and PEDOT:PSS/FMoS_2_ HTLs. In both cases, the thin films exhibited strong (100), (200), and (300) diffractions along the z axis, confirming the existence of the strong edge-on lamellae configuration of P3HT [63]. In addition, the absence of π–π staking peak (010), corresponding to the face-on orientation near the z axis, indicates that P3HT preferentially adopted the edge-on configuration in both PEDOT:PSS and PEDOT:PSS/FMoS_2_ HTLs. Since the use of –CH_3_ group-functionalized substrates tends to promote the face-on orientation of P3HT [55,56], we aimed to improve such configuration of the active layer by incorporating the described oleylamine (having –CH_3_ groups)-functionalized MoS_2_ into PEDOT:PSS, but we did not observe any significant difference in its molecular orientation, maybe because the low FMoS_2_ concentrations used were not sufficient for such change. Thus, we can conclude that the PCE and J_sc_ enhancement in the OCSs having PEDOT:PSS/FMoS_2_ as HTL may be due to its surface compatibility for the active layer deposition, as observed in the AFM and contact angle measurements. 

**Figure 6:**
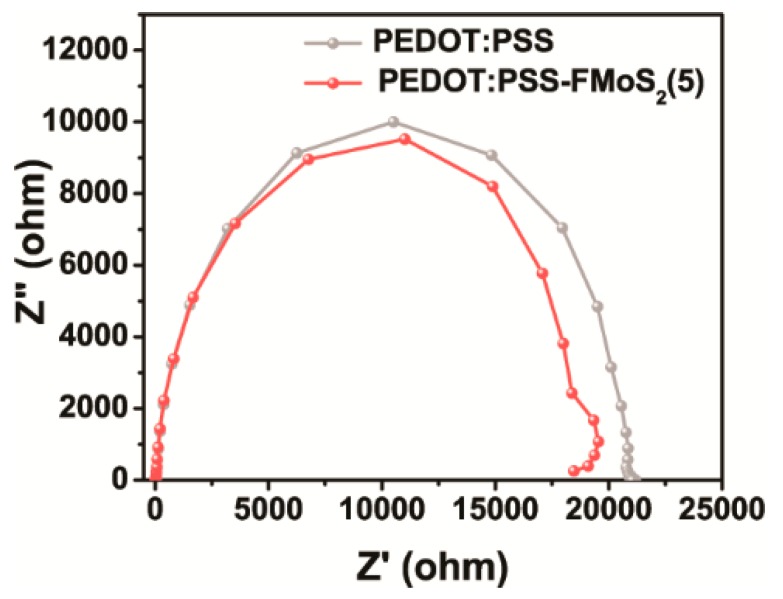
Electrical impedance spectra of organic solar cells based on poly(3,4-ethylenedioxythiophene):poly(styrenesulfonate) (PEDOT:PSS) and PEDOT:PSS/oleylamine-functionalized MoS_2_ (5 µL) (PEDOT:PSS/FMoS_2_(5)) as hole-transportation layer.

Electrical impedance spectroscopy (EIS) was performed to investigate the charge transport dynamics of the OSCs fabricated with PEDOT:PSS and PEDOT:PSS/FMoS_2_(5) as HTL (Figure 6). This analysis allowed us to observe the current response by applying alternating current voltage as a function of frequency; the OSCs with PEDOT:PSS/FMoS_2_(5) demonstrated slightly lower charge transfer resistance, revealing that the holes were effectively transported from the active layer to the anode (ITO). In order to elucidate the origin of the improvement in the photovoltaic performance, especially both FF and J_sc_ for PEDOT:PSS/FMoS_2_(5), we further calculated the resistance of the devices. In general, it is well known that lower series resistance (R_S_) and higher shunt resistance (R_SH_) are required to achieve higher FF in the solar cell device [64]. Based on the J–V curves obtained from the devices, it is clearly revealed that the device with PEDOT:PSS/FMoS_2_(5) as HTL showed the lowest R_S_ while maintaining higher R_SH_, leading to enhancement in charge extraction. The corresponding R_S_ value of cells employing PEDOT:PSS/FMoS_2_(5) as HTL was 134.8 Ω∙cm^2^, while the reference showed 180.0 Ω∙cm^2^. Lower R_S_ indicates that better interfacial contact and charge collection efficiency were obtained due to the addition of the conducting FMoS_2_ layer. In the case of the R_SH_, no significant changes in the shunt resistance were observed for the devices. In the point of view of the identical R_SH_, barrier resistance at the interface and the leakage current level flowing across the photoactive layer is similar. Therefore, the addition of FMoS_2_ might contribute to extract photoexcited charges efficiently by lowering the R_S_.

## 4. Conclusions

The application of solution-processed PEDOT:PSS/FMoS_2_ hybrids as effective HTLs for OSCs has been successfully demonstrated. Raman, UV-Vis, PL, TEM, and AFM analyses confirmed the successful exfoliation of bulk MoS_2_ into few-layer nanosheets in the presence of oleylamine via a simple and cost-effective solution-based method. The OSCs fabricated with the synthesized PEDOT:PSS/FMoS_2_ hybrids as HTL exhibited PCE values up to 3.74%, which is 15.08% higher than that of the reference cells having simple PEDOT:PSS as HTL. The hybrid HTL films showed better surface properties for the deposition of the hydrophobic active layer, consequently, the charge-transfer resistance was minimized for OSCs fabricated with hybrid HTL compared with reference cells, improving the OSC performance. Due to their simple preparation method, 2D FMoS_2_-incorporated PEDOT:PSS-based HTL provides valuable alternative HTL for OSCs. 

## Figures and Tables

**Table 1:** Photovoltaic performance of poly(3-hexylthiophene):[6,6]-phenyl-C61-butyric acid methyl ester-based organic solar cells having poly(3,4-ethylenedioxythiophene):poly(styrenesulfonate) (PEDOT:PSSS) and PEDOT:PSS/oleylamine-functionalized MoS_2_ (FMoS_2_) as hole-transportation layers.

FMoS_2_ Concentration (µL) in PEDOT:PSS	PCE (%)	V_oc_ (V)	J_sc_ (mA cm^−2^)	FF (%)
0 (Reference)	3.25 ± 0.03	0.671 ± 0.004	7.92 ± 0.09	61.2 ± 0.26
5	3.74 ± 0.02	0.665 ± 0.004	9.02 ± 0.17	62.24 ± 0.41
20	3.51 ± 0.15	0.667 ± 0.006	8.57 ± 0.33	61.34 ± 0.68
50	3.39 ± 0.08	0.669 ± 0.005	8.30 ± 0.17	61.10 ± 0.48

## Supplementary Materials

The following are available online at https://www.mdpi.com/2079-4991/9/9/1328/s1, Figures S1 and S2: PL spectra and AFM image of oleylamine-functionalized MoS_2_ (FMoS_2_) respectively, Figures S3 and S4: Contact angles and AFM images of PEDOT:PSS and PEDOT:PSS combined with FMoS_2_ respectively, Figure S5: (a) UV-Vis transmittance spectra of PEDOT:PSS and PEDOT:PSS combined with FMoS_2_, (b) UV-Vis absorbance spectra of P3HT:PCBM thin film spin-coated on PEDOT:PSS and PEDOT:PSS FMoS_2_.
