# Supplementary material for: Solution-Processed PEDOT:PSS/MoS2 Nanocomposites as Efficient Hole-Transporting Layers for Organic Solar Cells
Source: Nanomaterials (Basel). 2019 Sep 16;9(9):1328. doi: 10.3390/nano9091328 (PMC6781061; doi:10.3390/nano9091328)
Supplement: Supplementary file 1 [file nanomaterials-09-01328-s001.pdf]

# Electronic Supporting Information

## Solution-Processed PEDOT:PSS/$MoS_2$ Nanocomposites as Efficient Hole-Transporting Layers for Organic Solar Cells

Madeshwaran Sekkarapatti Ramasamy[a], Ka Yeon Ryu[a], Ju Won Lim[a], Asia Bibi[a], Hannah Kwon[a], Ji-Eun Lee[a], Dong Ha Kim[a]* and Kyungkon Kim[a]*

[a]Department of Chemistry and Nanoscience, Ewha Womans University, 52 Ewhayeodae-gil, Seodaemun-gu, Seoul 03760, Republic of Korea

* Corresponding author.

E-mail address: kimkk@ewha.ac.kr (Kyungkon Kim), dhkim@ewha.ac.kr (Dong Ha Kim).

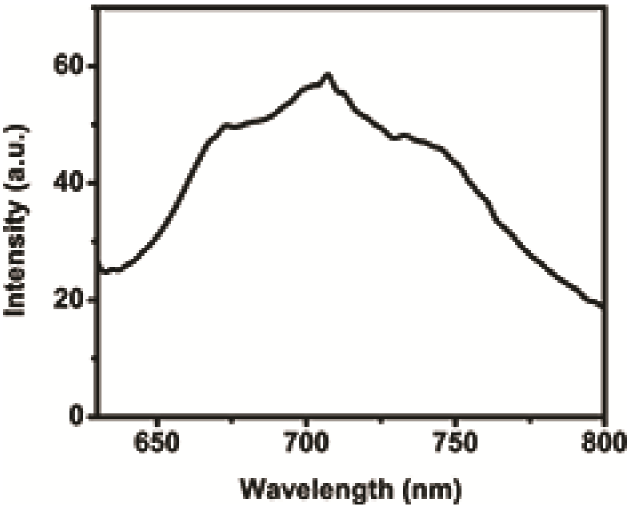


**Fig. S1.** Photoluminescence (PL) spectra of oleylamine-functionalized $MoS_2$ ($FMoS_2$).

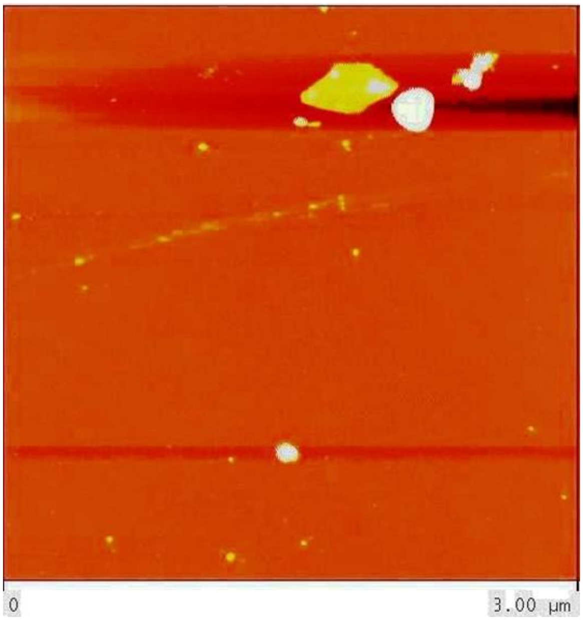


**Fig. S2.** AFM image of oleylamine-functionalized $MoS_2$ ($FMoS_2$).

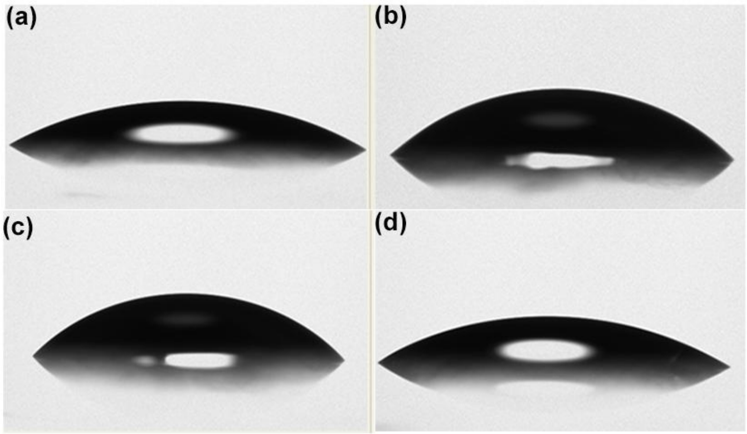


**Fig. S3.** Contact angles of (a) poly(3,4-ethylenedioxythiophene):poly(styrenesulfonate) (PEDOT:PSS) and PEDOT:PSS combined with (b) 5, (c) 20, and (d) 50 µl of oleylamine-functionalized $MoS_2$ thin films spin coated on ITO substrates.

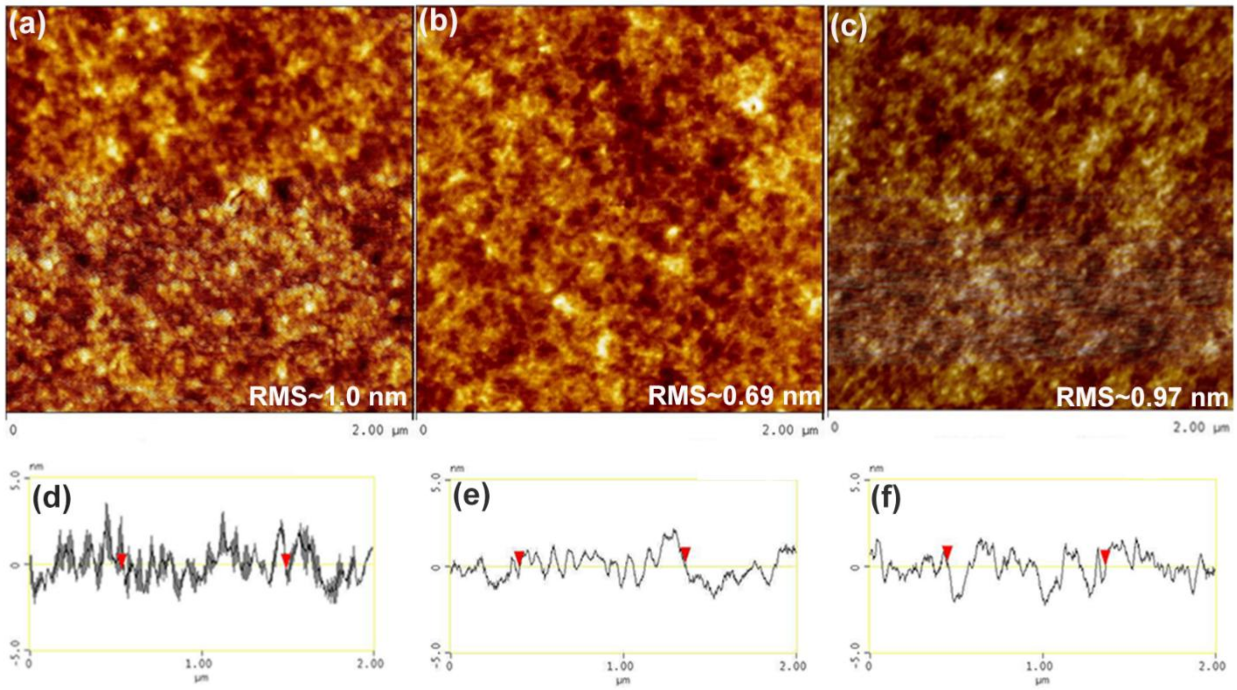


**Fig. S4.** AFM images of (a) poly(3,4-ethylenedioxythiophene):poly(styrenesulfonate) (PEDOT:PSS) and PEDOT:PSS combined with (b) 5, and (c) 50 µl of oleylamine-functionalized $MoS_2$.

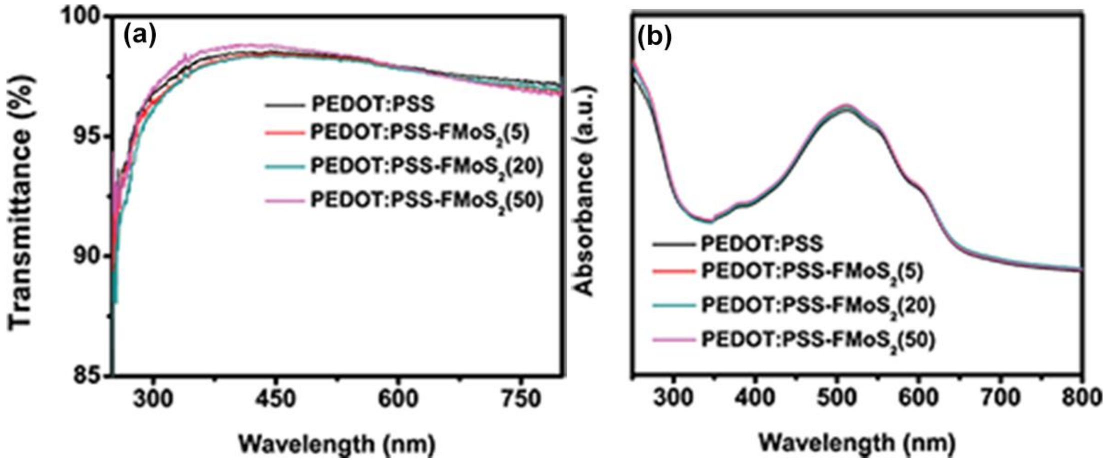


**Fig. S5.** (a) UV-Vis transmittance spectra of PEDOT:PSS and PEDOT:PSS combined with 5, 20, and 50 µl of oleylamine-functionalized $MoS_2$. (b) UV-Vis absorbance spectra of P3HT:PCBM thin film spin-coated on PEDOT:PSS and PEDOT:PSS combined with 5, 20, and 50 µl of oleylamine-functionalized $MoS_2$.
